# Histochemical and Immunohistochemical Identification of Mast Cells in the Rat Lacrimal Glands

**DOI:** 10.1111/vop.70005

**Published:** 2025-04-30

**Authors:** Ozkan Yavas

**Affiliations:** ^1^ Department of Veterinary Pathology Bursa Uludag University Bursa Turkey

**Keywords:** chymase, lacrimal glands, mast cells, rat, tryptase

## Abstract

**Objective:**

This study aims to investigate the presence, microanatomical localization, and immunohistochemical subtypes of mast cells in the lacrimal glands (LGs) of rats.

**Animals:**

A total of ten female Sprague–Dawley rats' LGs were evaluated in this study.

**Procedure(s):**

The experimental animals were euthanized. LG samples were collected and fixed in 10% neutral formaldehyde for 48 h. Sections were taken from the paraffin‐embedded samples and stained for microscopic examination. The histochemical properties of lacrimal gland epithelium and the composition of tears were examined by Periodic acid‐Schiff and Alcian blue staining and evaluated microscopically. Toluidine blue staining and tryptase and chymase immunohistochemically demonstrated localization and phenotypes of mast cells.

**Results:**

The existence and varying numbers of mast cells were successfully demonstrated in all three LGs by Toluidine blue staining, and the highest number of mast cells was observed in the extraorbital lacrimal gland, while their numbers were almost the same in the intraorbital lacrimal gland and Harderian gland. Immunohistochemically, chymase‐positive mast cells were more numerous than tryptase‐positive cells.

**Conclusions:**

This study is the first documentation of the presence of mast cells with different phenotypes in rat LGs.

## 1 Introduction

Vision is an extremely important biological event. Eye and accessory components, including lacrimal glands (LGs), are essential for vision. LGs are exocrine glands, and rats have three pairs of LGs in different locations relative to the globe [1, 2]. Harderian glands (HGs) are pigmented LGs located posterior to the eye. The intraorbital lacrimal gland (ILG) is located within the orbit at the caudolateral aspect of the HG. The extraorbital lacrimal gland (ELG) is the largest and is located anterior to the salivary gland at the base of the ear [1, 2]. Histologically, LGs consist of lobular structures separated from each other by thin, loose connective tissue. Each lobule has numerous basic functional units (acini) responsible for producing tears. Acini center on ducts that drain fluids onto mucosal surfaces. Fluid composition is unique to each LG. A thin connective tissue constitutes the outermost capsular layer of LGs [3].

The primary function of LGs is the production of tears, which are composed of various components such as water, proteins, electrolytes, lipids, mucin, and lysozyme. ILGs also produce pheromones for social behavior in the rat colonies [2]. To ensure a healthy ocular surface, LGs, and sebaceous glands provide tears and lipids to avoid excessive evaporation of the tear. Dysfunction of LGs results in a decrease and change in tear composition and loss of aqueous products required in ocular surface physiology and leads to many visual problems, such as keratoconjunctivitis sicca (KCS) [4, 5]. Local inflammatory changes and some systemic diseases, such as diabetes mellitus, impact LG function, and it is known that one of the most common diabetic complications on the ocular surface is KCS due to the impaired synthesis of the tear film [6].

Mast cells (MCs) are critical immune effectors and regulatory cells. They are derived from myeloid lineage and, following a short period in the peripheral circulation, they migrate to and reside in body locations in close contact with the external environment. The proximity of MCs to the external environment (i.e., skin, pulmonary, and gastrointestinal mucosa) is essential for a rapid response against various inflammatory stimuli [3]. MCs are crucial members of the defense system against many pathogens and allergens, with various mediators in their cytoplasmic granules and their phagocytic potential [7]. The phenotypes of MCs can be classified based on cytoplasmic enzymes, namely tryptase (+) (MCT) and tryptase‐chymase (+) (MCTC) MCs. This classification is important as it is known that different MC phenotypes predominate even in different microanatomical regions of the same organ and undertake different functions in these areas [8, 9]. The role of MCs in allergic reactions (type 1 hypersensitivity) is well‐known, and recent research has revealed their unique functions in a range of conditions, from cancer to fibrosis [9, 10, 11]. Tryptase is the most abundant and major secretory granule in many pathologies, from allergies to pain generation [12]. Another important one is chymase, and its role in many diverse pathobiologies has been documented [13].

MCs are more abundant in rats than in mice, especially in Wistar albino rats, when compared to Sprague–Dawley rats and these cells are more abundant in mesenteric lymph nodes, tongue, and sciatic nerves than in other lymph nodes, organs such as the heart, salivary gland, thymus, pancreas, and are almost absent in the kidney, pituitary gland, testis, bone, brain, optic nerve, and eye [14]. MCs are also located close to sensory nerve endings, and their activation or inhibition can modulate the excitability of sensory receptors and tear production. Little is known about the existence and microanatomical distributions of MCs in the rat LGs. Wood et al. [3] examined the presence of MCs in the ELGs of rats histochemically. However, immunohistochemical identification of MCs according to their enzyme content was not performed in the aforementioned study. Since enzymes drive the diverse functions of MCs, this descriptive study aimed to document, for the first time, the immunohistochemical MC phenotypes in the LGs of Sprague–Dawley rats *(Rattus norvegicus)*.

## 2 Methods

### 2.1 Animals

Ten female Sprague–Dawley rats were obtained from the Experimental Animals Breeding and Research Center of Bursa Uludağ University. They were housed at 20°C–22°C with 60%–70% humidity in a controlled room set to a 12‐h light/dark cycle and had access to standard rat chow and water *ad libitum*. LG samples were obtained from control animals from another study (Ethical approval number: 2020‐03/04) and had not been subjected to any surgical or therapeutic intervention. These samples were collected after the sevoflurane (Sevorane %100, Italy) euthanasia of the control animals.

### 2.2 Histopathological Examination

Tissue samples were fixed in 10% formaldehyde solution for 48 h and transferred to tissue cassettes, dehydrated in an ascending series of ethanol, cleared in xylene, embedded in paraffin, cut into 4‐μm serial sections, and sections were placed onto Poly‐L‐Lysine coated slides. To evaluate the histological characteristics of the glands, all sections were stained with Hematoxylin–Eosin (H&E), Periodic Acid Schiff (PAS), and Alcian Blue (AB). Goblet cells in rat gastrointestinal sections were used as positive controls in PAS and AB staining. In PAS and AB staining, glands were classified according to staining intensity: no staining, +: weak staining, ++: moderate staining, and +++: strong staining and summarized in Table 1.

**TABLE 1:** Comparison of histochemical and immunohistochemical staining among the three lacrimal glands (LG, ILG, and HG).

	Extraorbital lacrimal gland (ELG)	Intraorbital lacrimal gland (ILG)	Harderian gland (HG)
Alcian blue	+	+	—
PAS	+	+	—
Toluidine blue^a^	96.8	6.4	6.8
Tryptase	+++	+	+
Chymase	++++	++	++

*Note:* For histochemical stains (PAS and AB); +, weak positive; ++, moderate positive; +++, strong positive and for immunohistochemical staining (tryptase and chymase); +, very low; ++, low; +++, moderate; ++++, high; +++++, very high.

^a^ Average number of TB+ cells over 5 fields under ×100 magnification.

Metachromatic properties of MCs were visualized by Toluidine Blue staining. The presence and location of MCs were examined using a light microscope in toluidine blue‐stained sections. Toluidine blue positive MCs were counted at 100× magnification, and results were presented as the average number over 5 counted fields. Rat skin sections were used as positive controls in TB staining.

### 2.3 Immunohistochemical Examinations

Sections prepared from formaldehyde‐fixed paraffin‐embedded tissue samples were immunostained to phenotypically classify MCs as tryptase and chymase positive. After deparaffinization in xylene, slides were rehydrated through graded alcohol and rinsed in distilled water. Endogenous peroxidase activity was blocked with 3% hydrogen peroxide in methanol. Samples were boiled in a citrate buffer (pH 6) in a microwave for 10 min at 600 W for antigen retrieval. After protein blocking, slides were incubated with the primary antibodies (tryptase 1:100 dilution, Santa Cruz Biotechnology, USA, sc‐59587; chymase 1:100 dilution, biorbyte, United Kingdom, orb‐58947) at +4 ^ο^C overnight and later with a horseradish peroxidase polymer using DAB kit protocol (Ultra Large Volume, Thermo Fisher, United Kingdom). To visualize the antibody‐enzyme complex, 3, 3′‐diaminobenzidine tetrahydrochloride (DAB) was used. Slides were counterstained with Mayer's hematoxylin and examined by light microscopy. Granulated cells showing brown staining with tryptase and chymase antibodies were considered positive, and their localization and immunophenotypes in all three LGs were examined under light microscopy. Cytoplasmic brown staining in MCs was considered specific for immunohistochemistry, as positive control slides from rat skin samples showed similar staining characteristics.

## 3 Results

### 3.1 Histological Examination

**FIGURE 1:**
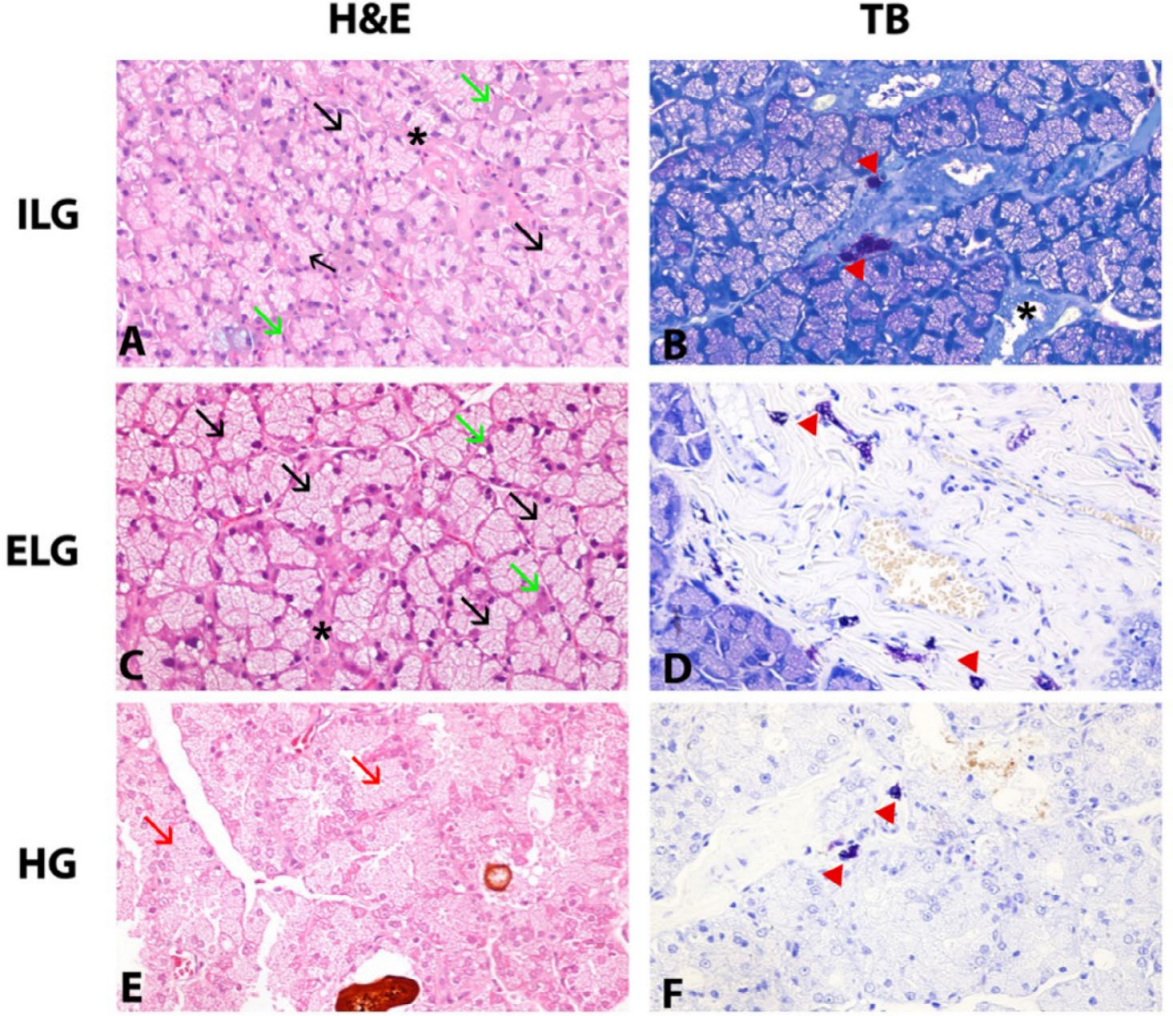
Histologic appearance of lacrimal glands and distribution of mast cells in lacrimal glands. (A) Intraorbital lacrimal gland lined with mucopolysaccharide‐secreting mucous secretory cells, Hematoxylin & eosin staining, magnification ×40. (B) Mast cells observed in the interlobular interstitial space of the intraorbital lacrimal gland, Toluidine blue staining, magnification ×40. (C) Tubuloacinar structures and ducts composed of mucous acini in the extraorbital lacrimal gland, Hematoxylin & eosin staining, magnification ×40. (D) Numerous mast cells in the interlobular interstitial space, blood vessel and around the acini in the extraorbital lacrimal gland, Toluidine blue staining, magnification ×40. (E) Serous acini in Harderian glands, perforin in the center of acinus. Hematoxylin & eosin staining, magnification ×40. (F) Mast cells observed in the interlobular interstitial space and around the acin of Harderian gland, Toluidine blue staining, magnification ×40. Black arrow: mucous acinus, red arrow: serous acinus, asterisk: ductus, green arrow: myoepithelial cell, red arrowhead: Mast cell.

Hematoxyline and eosin (H&E) stained sections of LGs were within normal limits with no evidence of degeneration, inflammation, and proliferative changes. The acini in the ILGs contained mucous acini, distinguished by their columnar pale‐stained and foamy cytoplasm secreting mucopolysaccharide. Histochemical analysis of the mucin content showed weak staining with alcian blue and negative staining with PAS. Secretory ducts located between the acinar structures were easily identified. Myoepithelial cells were observed close to and parallel to the basement membrane of acinar cells. ELGs were mostly tubuloacinar with narrow lumens and ducts and contained mainly mucous acini. (Figure 1A).

Acini showed weak staining with PAS and alcian blue. The acinar cells were surrounded by myoepithelial cells, as in ILG (Figure 1C).

The acini of the HGs were lined with serous secretory cells with numerous prominent cytoplasmic vacuoles and round nuclei located on the basal side of the cells, which stained negatively with PAS and AB. Porphyrin, a red‐brown pigment, was distinguished in the lumen of some acini. The myoepithelial cells surrounding the acini and between the secretory cells and the basement membrane were not easily distinguished on slides (Figure 1E) (Table 1).

### 3.2 Toluidine Blue Staining

MCs were detected in all three LGs and were identified microanatomically in the subcapsular areas, around the acini, interlobular interstitial tissue, and the periductal areas. In these locations, both granulated (intact) and degranulated (active) MCs were detected. These cells were found to be located in close proximity to blood vessels and peripheral nerves. ELGs were found to have a much higher number of MCs than ILGs and HGs (average number 96.8, 6.4, and 6.8, respectively) (Figure 1B,D,F) (Table 1).

### 3.3 Immunohistochemical Staining

Tryptase and chymase‐positive MCs were demonstrated immunohistochemically. In some MCs, nuclei were not visible due to the intensity of the staining. Due to degranulation, small spots of free brown staining were also observed in some extracellular spaces around MCs.

**FIGURE 2:**
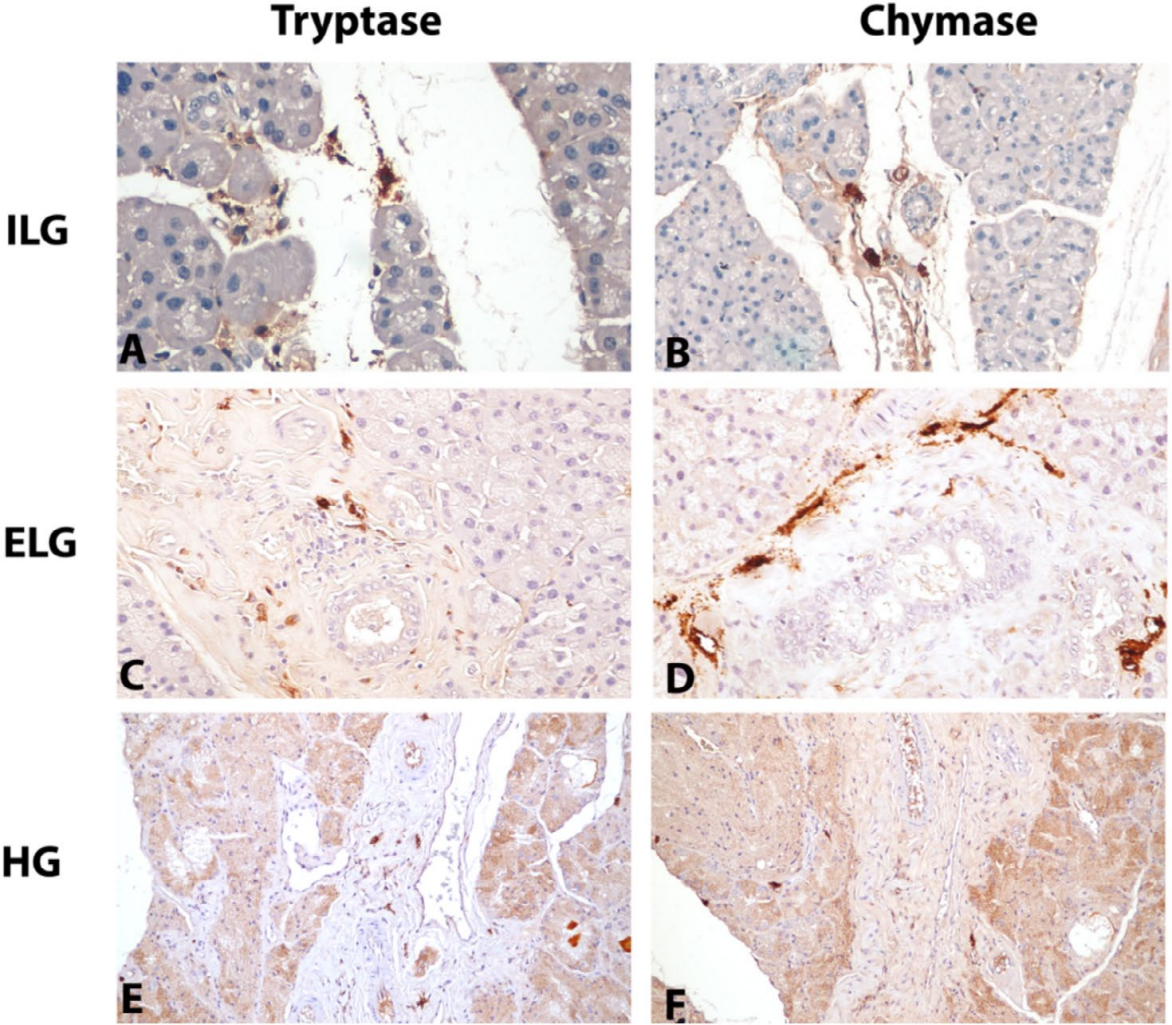
Immunohistochemical analysis of phenotypic characteristics of mast cells in lacrimal glands. (A, B) Tryptase‐ and chymase‐positive mast cells in the intralobular lacrimal glands in the interlobular space and around the vessels, magnification ×40. (C, D) Tryptase and chymase positive mast cells in the interlobular interstitial space and around acini in the extra orbital lacrimal gland, magnification ×40. (E, F) Demonstration of a small number of tryptase and chymase positive mast cells in the interlobular interstitial space and between acini in the Harderian gland, magnification ×20.

Chymase‐ and tryptase‐positive MCs in the ILGs were generally observed in the interstitial tissue and around blood vessels. A small number of chymase and tryptase‐positive MCs were found in the ILG (Figure 2A,B). The number of MCs in the ELG was significantly higher than in the other two LGs. These cells were found less around the acini and periductal areas and more in the interlobular interstitial tissue. Chymase‐positive MCs were observed more than tryptase‐positive MCs. Their number was considerably lower than in ELG. In the ELG, the number of chymase‐positive MCs was higher than the number of tryptase‐positive MCs (Figure 2C,D). Chymase and tryptase‐positive MCs were observed in the HG. The few MCs observed were in the interstitial tissue (Figure 2E,F). Overall, the number of chymase‐positive MCs was higher than that of tryptase‐positive MCs in all three LGs (Table 1).

## 4 Discussion

In the present study, it was observed that ILGs and ELGs were tubuloacinar structures and contained secretory units and draining ducts. The ducts had lumens of different sizes. However, we classified all these structures as secretory ducts, as Wood et al. [3], without defining whether the ducts were intralobular or interlobular. In the acini of ILGs, mostly serous cells were detected, whereas in ELGs, both serous and mucous cells were detected. In both extraorbital lacrimal and ILGs, acini were surrounded by a thin layer of myoepithelial cells. In our study, the general histologic structures of the glands were similar to those previously reported by others [3, 15].

One of the LGs' most important functions is maintaining tear film homeostasis [16]. The corneal and scleral surface is covered with a three‐layered film of tear fluid, which is needed for protection and nutritional requirements. The tear film is constituted by the inner mucous, middle aqueous, and outer lipid layer [17]. Mucin in the innermost layer is produced by acinar cells of the LGs. Two types of mucins, neutral and acidic, are found in the tear film composition [18]. Allen et al. 1972 reported that acidic and neutral mucins were produced in both acinar and ductal epithelial cells [19].

Additionally, Wood et al. [3] reported that neutral mucins are secreted only by glandular epithelium. We used AB and PAS staining to demonstrate neutral and acidic mucins. Similar to previous studies, we found that neutral and acidic mucins were produced in acinar and ductal epithelial cells of ELGs.

MCs are found in most healthy tissues and organs in rodents. Their presence has also been reported in LGs, but species‐specific differences are observed [14]. Comparing MCs in LGs of experimental animals, MCs were found to be in high numbers in rat and mouse LGs compared to a rabbit. Although MCs in human LGs are reported to increase allergic reactions and disease, the number and localization of MCs in healthy LGs are not yet known [15, 20, 21]. In rats and mice, the number of MCs and lymphocytes in the LG increases with age [20, 21]. In contrast, the presence of MCs has not been reported in studies of aging humans [15].

The submitted study documented the phenotypes of MCs in the rat LGs for the first time. MCs have important functions in adaptive and innate immunity and their role in allergic reactions. The interface regions between the host and external environment have abundant MCs with functional plasticity, and the cytoplasmic content of MCs serves this plasticity. MCs are abundant at these interface sites, such as oral, nasal, and gastrointestinal mucosa, and are the first cells to respond to invading pathogens with their various cytokines and proteases [22]. Therefore, MCs and their functions are indispensable in a system, such as the eye and its accessories, which are in direct contact with the external environment and are constantly exposed to pathogens.

In addition to their important roles in allergic immune responses, MCs are believed to be multifunctional key effector cells in various pathological conditions [22, 23, 24], from acute inflammation to chronic fibrotic disease [25, 26, 27]. Due to their preformed and de novo synthesized cytokines and proteases, MCs contribute to acute and chronic inflammatory responses [15]. MCs are not identical in all tissues, and this heterogeneity or plasticity may result in diverse effects in different organs due to interactions with local cells [28]. Garcia‐Rodriguez and colleagues [29] demonstrated that MCs may develop and exhibit individualized immune responses to meet tissue requirements against various bacteria in different body locations. Hence, knowledge of the presence and phenotypes of MCs in LGs may help to understand their contribution to various physiological and pathological processes related to vision. In this context, we propose that MCs may play essential roles in managing local oculoglandular infections, and more detailed studies are needed.

Multifunctional tryptase and chymase enzymes are major proteins synthesized and stored by MCs. Thanks to these serine proteases, MCs display proinflammatory and anti‐inflammatory features in mice [30, 31]. Xu et al. [31] reported that MCs protect mice from mycoplasmal infections through tryptase and chymase enzymes. A recent study by Fujita et al. [32] showed increased chymase‐positive MCs and mediation of inflammatory processes in the LGs with IgG4‐related ophthalmic diseases. In the present study, chymase‐expressing MCs were significantly more numerous than tryptase‐expressing MCs. Since chymase expression is observed more in IG4‐related ophthalmic diseases and the eye is often affected by allergic disease, this may explain why chymase is expressed more in LGs. Therefore, examination of MCs in the LGs will provide basic data on the inflammatory conditions of the oculoglandular system.

MCs have been shown to increase on the ocular surface immediately after ocular damage (within 1 h), followed by large numbers of neutrophils [33]. Another study demonstrated the release of IL‐33 from the corneal epithelium following damage to the ocular surface, activation of MCs by IL‐33, and subsequent recruitment of large numbers of neutrophils into the site of injury [34]. Thus, lacrimal MCs may have pivotal potential to modulate oculoglandular inflammation. More MCs in the ELG compared to other LGs may suggest that these glands are more involved in inflammatory conditions, but more studies are needed to explain this observation.

The number of studies investigating the presence and function of MCs in LGs is quite limited. Williams et al., 1994 displayed MC localization and changes in their numbers with age in the LGs of rats. In the same study, the authors reported that MCs were localized around blood vessels adjacent to acinar structures, interlobular connective tissue, and capsular connective tissue [34]. The microanatomical location of MCs in our study was similar to that described by Williams et al. 1994. However, our study added to the findings of Williams et al. by providing data from immunohistochemical phenotyping of MCs according to the presence of tryptase and chymase. This classification is extremely important since types and amounts of enzymes are known to cause functional differences in MCs in different organs and even in different microanatomical parts of the same organ. A limited number of fresh studies have questioned the possible role of MCs in ocular diseases. In a very recent study, the potential effects of MCs and chymase in IgG4‐associated ophthalmic disease were reported, and it was revealed that chymase regulates fibrotic changes in LGs via TGF beta‐1 [32]. Another study reported an increased number of MCs in the LGs with advancing age and age‐related structural changes in wild‐type and MC‐deficient rats [35].

This study is the first to examine phenotypes of MCs in all three LGs of rats using commercially available antibodies. The present study successfully identified tryptase (+) and chymase (+) MC phenotypes with varying numbers in all three LGs.

## 5 Conclusions

This study described the histochemical features of the LGs and the distribution and phenotype of MCs in these glands. Since MC proteases have beneficial and detrimental roles in ocular health, the demonstration of lacrimal MCs and their phenotypes may bring a new perspective to understanding and managing ocular diseases.

## Author Contributions

**Ozkan Yavas:** conceptualization, data curation, formal analysis, investigation, methodology, writing – original draft, writing – review and editing.

## Ethics Statement

This study protocol was reviewed and approved by Bursa Uludag University Local Ethics Committee, approval number [2020‐04/03].
